# Ultrasound- and Temperature-Induced Gelation of Gluconosemicarbazide Gelator in DMSO and Water Mixtures

**DOI:** 10.3390/gels3020012

**Published:** 2017-04-18

**Authors:** Mothukunta Himabindu, Aruna Palanisamy

**Affiliations:** Polymers and Functional Materials Division, Indian Institute of Chemical Technology (CSIR-IICT), Hyderabad, Telangana 500007, India; binduhimachemistry@gmail.com

**Keywords:** amphiphilic gelators, ultrasound, thermoreversible, hydrogen bonding, gluconohydrazide

## Abstract

We have developed amphiphilic supramolecular gelators carrying glucose moiety that could gel a mixture of dimethyl sulfoxide (DMSO) and water upon heating as well as ultrasound treatment. When the suspension of gluconosemicarbazide was subjected to ultrasound treatment, gelation took place at much lower concentrations compared to thermal treatment, and the gels transformed into a solution state at higher temperatures compared to temperature-induced gels. The morphology was found to be influenced by the nature of the stimulus and presence of salts such as KCl, NaCl, CaCl_2_ and surfactant (sodium dodecyl sulphate) at a concentration of 0.05 M. The gel exhibited impressive tolerance to these additives, revealing the stability and strength of the gels. Fourier transform infrared spectroscopy (FTIR) revealed the presence of the intermolecular hydrogen bonding interactions while differential scanning calorimetry (DSC) and rheological studies supported better mechanical strength of ultrasound-induced (UI) gels over thermally-induced (TI) gels.

## 1. Introduction

The synthesis of low molecular weight building blocks from renewable resources for the development of soft [1,2] materials through supramolecular concept is of great interest. The products of this synthesis include many attractive properties, such as their inherent biodegradability, non-toxicity, and eco-friendliness with structural diversity, all of which lend them wider applications in chemistry, biochemistry and material sciences. To date, several methods have been employed to synthesize effective hydro and organogels by utilizing renewable plant-derived sources through supramolecular concept. Carbohydrate centered low-molecular weight gelators (LMWGs), which can be functionalized explicitly to yield biocompatible gels through a self-assembly process, find many biomedical applications [3,4]. There are also many reports on low molecular weight sugar-based amphiphilic [5,6] and bola amphiphilic gels [7] in which sugar polar head groups are held by linkages such as ester, ether, amine and urea between the hydrophilic head and hydrophobic tail groups in order to facilitate well-defined and stable macroscopic structures by secondary interactions [8,9,10,11,12,13]. They form a physically crosslinked network by self-aggregation through hydrogen bonding, π–π stacking, solvophobic interactions and van der Waals interactions [14,15,16,17,18,19,20]. The dynamic reversible nature of these interactions depends on the gelator structure, which gives these LMWGs the inherent ability to exhibit phase transition changes triggered by external stimuli, including ultrasound [21], temperature [22], pH [23], and light [24]. These materials have attracted increasing attention due to their wide applications in drug release [25], sensors [26], and catalysis [27].

Among these stimuli, low frequency ultrasound is a powerful therapeutic tool used in molecular imaging and in releasing drug molecules in tumor-targeted regions [28]. To initiate the aggregation of molecules that leads to the formation of highly stable 3D network through ultrasound stimuli, balancing hydrogen bonds with other physical interactions is the prerequisite for the gelling action. This induces changes in the morphology, and the orientation of microscopic surface features such as surface wettability [29,30,31]. Ultrasound is a novel method that often influences the mode of molecular self-assembled morphologies due to change in hydrogen bonding from intramolecular to intermolecular interactions [32]. Jiaju Xu described CuBuPc organogel formation assisted by ultrasound treatment and the fabrication of transistors by the doctor blading technique, which significantly increased the charge carrier mobility due to stronger π–π interactions providing a way for the development of electronic devices [33]. Dou et al. described a urea-based gelator which dissolved in polar aprotic solvents at elevated temperatures, in which a precipitate was generated upon cooling the hot solution to room temperature. Further gels were obtained by sonicating the hot solution in a water bath for 10 s [34]. Navneet Goyal et al. have explored a series of sugar-derived pH responsive gelators, and studied the Naproxen release kinetics by entrapping this non-steroidal anti-inflammatory drug in a dimethyl sulfoxide (DMSO)/water mixture gel under neutral and acidic conditions [35].

In this contribution, we report the synthesis and gelation properties of a thermoreversible amphiphilic sugar-based gelator in a mixture of H_2_O/DMSO (20/80 *v*/*v*). DMSO is a polar organic solvent miscible with water and other aqueous solvents. DMSO is used as a drug vehicle both in vivo and in vitro experiments. It is also used as an effective alternative cancer cure. Hence, the gelation of a mixture of DMSO and water finds many applications in the area of drug release. The amphiphilic gelator, with a hydrophilic glucose moiety as the head group and a hydrophobic tail connected by polar semi carbazide linkages, was able to immobilize a mixture of polar aprotic solvent and water. More interestingly, different morphologies were observed for the gels obtained by heat-cooling method and ultrasound treatment-resting method. The head group with multiple hydroxyl groups and a tail end comprising of alkyl chains tagged through urea-type linkages self-aggregates into continuous folded sheets. A combination of spectroscopic methods, X-ray diffraction technique and microscopic methods were used to arrive at a hypothesis for the molecular arrangement of the gels induced by heat and ultrasound.

## 2. Results and Discussion

**Figure 1:**
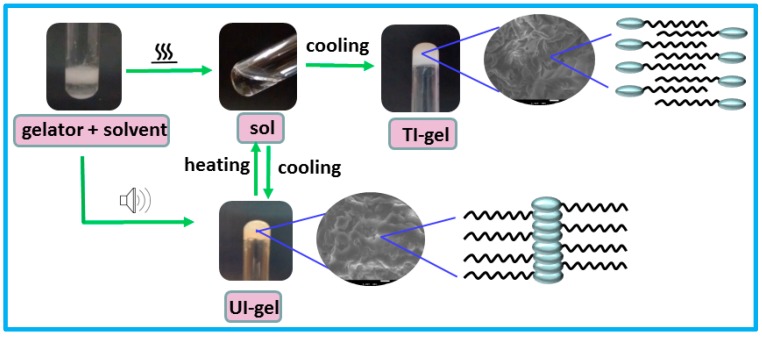
Digital photographs of sol gel transition of ultrasound- and thermally-induced gels.

The gelator was found to gel a mixture of polar aprotic solvents DMSO, NMP (*N*-methyl 2-pyrrolidone), DMF (*N*,*N*-dimethyl formamide), DMAc (*N*,*N*-dimethyl acetamide) and water at a concentration of 20–25 mg/mL at (80/20 *v*/*v*) (Table S1). However, we have specifically chosen the DMSO/water gels for further studies since DMSO has already been reported to be used as a drug vehicle. The gel obtained by the ultrasound-induced (UI-gels) was stable at room temperature and upon heating it transformed to a solution state. This hot solution converted to a gel upon cooling to ambient temperature. The ultrasound-induced gelation occurred at relatively less concentrations and gelation time than the thermally-induced gelation (TI-gels), facilitating the formation of continuous folded sheets as shown in Figure 1. Gelation occurred in the presence of salts such as NaCl, KCl, CaCl_2_ and SDS (sodium dodecyl sulphate) at 0.05 M concentration. The presence of different ions in the solvent led to an increase in the gelation time [36].

### 2.1. Fourier transform infrared spectroscopy (FT-IR)

**Figure 2:**
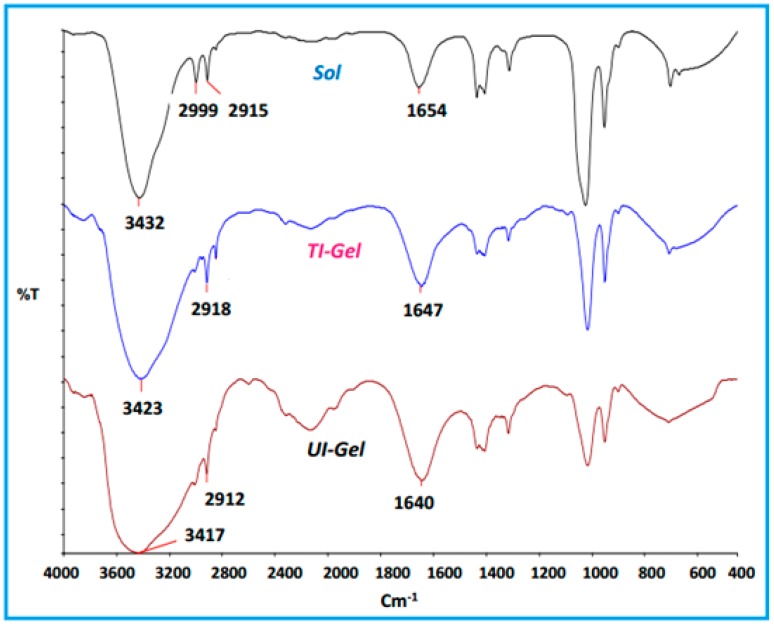
FTIR spectrum of sol, thermally-induced (TI) gel and ultrasound-induced (UI) gel of the gelator in dimethyl sulfoxide (DMSO)/water mixtures (80/20 *v*/*v*) at a concentration of 30 mg/mL.

Low molecular weight gelators self-assemble into a three-dimensional network and restrict the flow of bulk fluid. The driving forces responsible for the three-dimensional network is built by intermolecular interactions like hydrogen bonding in most of the urea-type molecules. To probe the change in hydrogen bonding interactions with respect to the nature of the stimuli, IR spectra were recorded for the TI-gels and UI-gels of the gelator. The spectrum of the gelator (Figure 2) in the solution phase exhibits peaks at 3432 cm^−1^ (NH stretching frequency) and 1654 cm^−1^ (>C=O stretching frequency) ascribed to non-hydrogen bonded NH and carbonyl stretching frequencies. In the gel state, the NH band shifted to 3423 cm^−1^ (TI-gels) and 3417 cm^−1^ (UI-gels) and the carbonyl band shifted to 1647 cm^−1^ (TI-gels) and 1640 cm^−1^ (UI-gels), confirming the involvement of hydrogen bonding between >C=O and amide N-H groups in the gel state. In the case of the UI-gels, the extent of shifting of peaks was more compared to the TI-gels, indicating a greater extent of H bonding in the UI-gels.

### 2.2. Thermal Properties

**Figure 3:**
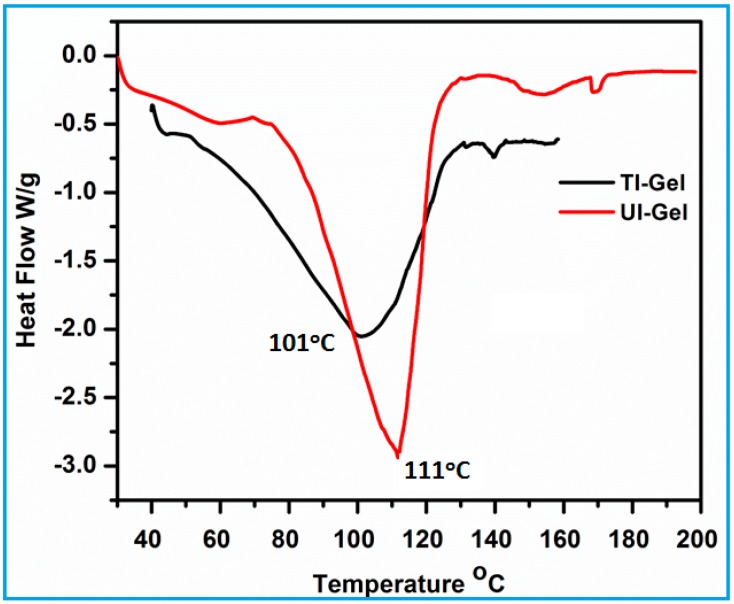
Thermogram of UI-gel and TI-gel in DMSO/water (80/20 *v*/*v*) at 30 mg/mL concentration.

To understand the thermal stability of the amphiphile in the self-assembled phase, differential scanning calorimetry (DSC) was employed to study the phase transition of the gels. The transitions are dependent on the concentration of the gelator, as well as the nature of the external stimuli. The DSC curves of TI-gel and UI-gel are presented in Figure 3, which shows a marked difference in the melting temperature of the both the gels. Thermal-induced gel exhibits a gel melting temperature at 101 °C, whereas UI gel shows transition at 111 °C. The higher melting temperature in case of UI-gel may be because of the mode of aggregation of the molecules through intermolecular hydrogen bonding interactions between hydrophilic head groups. These gels require more heat to disrupt the physical crosslinks in the gel phase, which are strengthened by secondary interactions. In case of thermally-induced gels, it is assumed that the alkyl chains are oriented and stacked one above the other and the energy required for disrupting the interactions is less. Thus, the nature of the stimuli has a dramatic influence on the aggregation behaviour and melting of the gels.

### 2.3. Morphology

**Figure 4:**
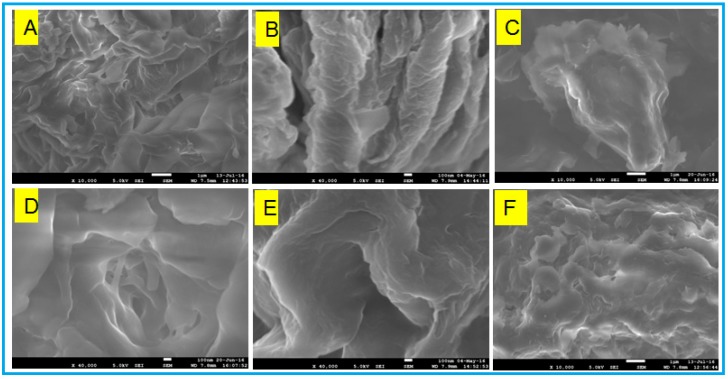
SEM images of xerogels of Gluconosemicarbazide gelator in DMSO/water mixture at a concentration of 30 mg/mL under different stimuli: (**A**) thermally-induced (1 µm); (**B**) SDS (100 nm); (**C**) KCl (1 μm); (**D**) NaCl (100 nm); (**E**) CaCl_2_ (100 nm); (**F**) ultrasound-induced (1 µm).

To estimate the microstructural changes of molecular aggregation upon sonication, heating, the influence of salts like KCl, NaCl, and CaCl_2_ at a concentration of 0.05 M, and surfactant viz. sodium dodecyl sulphate (SDS), morphological analysis was carried out by Scanning Electron Microscopy (SEM). SEM images (Figure 4) of the the xerogels of DMSO/water mixtures obtained from thermal induction showed continuous folded sheets of aggregates in a 3D network, and some of them are comprised of entangled and fused networks of sheets. Micrographs of ultrasound-induced xerogels shows a flake-like morphology. During the sonication process, the amphiphilic molecules arrange in a perfect order to enhance intermolecular hydrogen bonding interactions. This distinct microlevel morphology suggests that sonication plays a key role in directing the aggregates. It is known that presence of salts in the gelator favours the formation of charges on the head group of amphiphiles by reducing the electrostatic repulsions between the head groups, rendering the solubility in aqueous solvents [37]. However, the presence of small concentrations of salts and surfactants did not bring any considerable morphological changes in which the polar head groups favoured tight packing of the molecules through different modes of aggregations, leading to such distinct microscopic features [38,39]. Instead, the gels were found to be stable and their morphology intact with the addition of salts, an attractive property of the gels since stability of the gels in biological fluids is essential for biomedical applications.

### 2.4. Rheological Properties

To gain further insight into the dynamic mechanical properties of the gelators, at a constant temperature (25 °C) TI-gels and UI-gels were subjected to continuous frequency sweep and amplitude sweep to confirm their viscoelastic properties.

**Figure 5:**
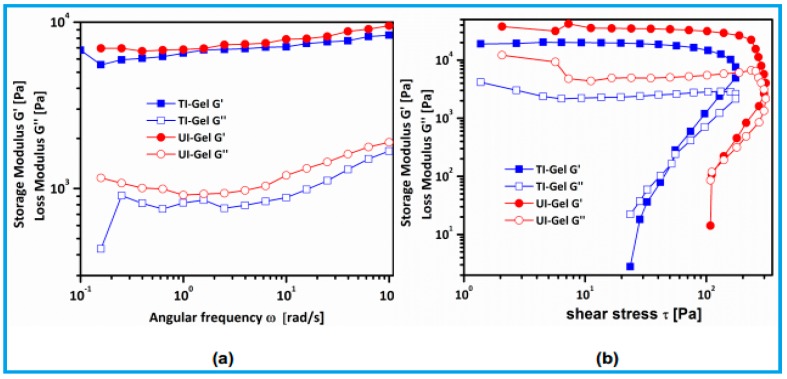
Storage modulus *G*′ and loss modulus *G*″ as a function of (**a**) angular frequency; (**b**) shear stress for gels at a concentration of 30 mg/mL.

In the frequency sweep measurements at a fixed amplitude of stress, both storage modulus (*G*′) and loss modulus (*G*″) of the UI-gels were found to be greater than those of the TI-gels, with linear response over the entire plateau region. It is evident from Figure 5a that storage modulus and loss modulus is invariant for all of the systems in entire frequency range with *G*′ > *G*″, suggesting the gel behavior and the stability of the system. Furthermore, to analyze the strength and stability of the ultrasound-induced and thermal-induced gels, *G*′ of the UI-gel is found to be greater than that of *G*′ of the TI-gel. This is due to the reinforcing action of intermolecular hydrogen bonding interactions induced by sonication [40].

In the amplitude sweep experiments, shear stress is applied to the sample sinusoidally. The response of the stress is shown in Figure 5b, where storage modulus G′ > G″ (loss modulus) during the initial application of shear stress. Both the gels were found to withstand lower stresses at which storage modulus exceeds loss modulus, and upon increasing the shear stress the magnitude of *G*′ starts decreasing with the reduction in the stored energy and begins to flow. At this stage the networks in the gel phase suffer a collapse by which gel disruption arises, and *G*′ subsequently crosses over *G*″ with *G*″ > *G*′ referred to as yield stress. The behavior of the gels in the vicinity of yield stress indicates the transition from gel state to sol state. Ultrasound-treated gel has a higher yield stress (σy = 108 Pa) than the thermally-induced gel (σy = 23 Pa). This implies that sonicated gel has higher mechanical strength than thermally-treated gel [41]. These results are consistent with DSC and SEM data, as discussed earlier.

### 2.5. Mode of Packing in the Xerogels

**Figure 6:**
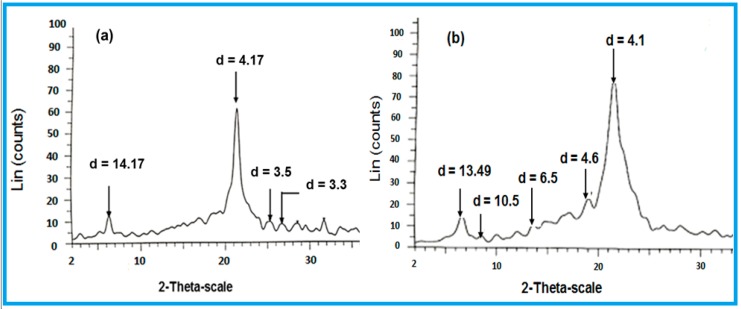
X-ray diffraction spectrum of xerogels of (**a**) thermally-induced gel and (**b**) ultrasound-induced gel.

To obtain deep insight into the aggregation mode arising from both sugar moiety and alkyl chains, wide angle XRD was employed. Figure 6a,b show the X-ray pattern of the gelator in the xerogel state, prepared from the DMSO/water mixtures under different conditions i.e., ultrasound and thermal treatments (Figure 6a), from freeze-thawing method at a concentration of 30 mg/mL. A very intense signal at 4.1 Å in both of the gels corresponds to the plane of the hydrogen bonding [42]. The diffractogram (Figure 6b) of the gelator obtained after ultrasound irradiation shows the series of peaks at 13.4, 6.5, and 4.6 Å, following a ratio of 1:1/2:1/3, which suggests that molecules are arranged in a layered structure in the gelator. The Braggs distance in the low angle region at the d spacing 13.49 and 14.17 Å (UI- and TI-gels) corresponds to extended molecular length of the gelators in which the molecules are arranged in bilayer organization by intra and interlayer hydrogen bonding and electrostatic interactions. Based on the type of aggregation, the molecular length of the self-assembled gelators would vary as reflected in the different d spacing values. A lower value is expected for UI-gels, due to the association of molecules through head group stacking. In TI-gels, interdigitated arrangement is expected in addition to the aggregation of head groups as proposed by John et al. [2,43]. In Figure 6a, peaks at 3.5 and 3.3 Å are assigned to the distance between the alkyl chains within the molecules and distance between the molecules along the hydrogen bonding plane, which is not found in case of the ultrasound-induced gelator suggesting that alkyl chains are loosely packed in the aggregates with head-to-head arrangement, as shown in Figure 1.

## 3. Conclusions

In this study, we report gluconosemicarbazide gelator with a hydrophilic glucose head and a hydrophobic fatty acid tail which could gelate a mixture of DMSO and water (80/20 *v*/*v*) both upon heating and ultrasonication. The thermally-reversible gels thus obtained were found to be stable in the presence of 0.05 M KCl, NaCl, CaCl_2_ and surfactant (sodium dodecyl sulphate). The different modes of aggregation arising due to the stimuli-induced gelation gives rise to different morphologies and behavior. The extent of intermolecular hydrogen bonding in ultrasound-treated gels is more compared to thermally-induced gels as inferred from FTIR, differential scanning calorimetry and rheological studies. The rheological studies also confirmed that the UI-gels are stronger than the TI-gels due to higher gelator-gelator intermolecular hydrogen bonding interactions which impart high strength that facilitates less dissipation of energy during the shear process.

## 4. Experimental

### 4.1. Materials

Glucono δ-lactone was purchased from Sigma Aldrich (Milwaukee, WI, USA). Hydrazine hydrate, octanoyl chloride, decanoyl chloride, lauryl chloride, myristoyl chloride, palmitoyl chloride, methanol, sodium azide and benzene were procured from SDFCL, Mumbai, India.

### 4.2. Synthesis of Gluconohydrazide

Delta gluconolactone (5 g, 1 equiv. is dissolved in 80 mL of methanol, and hydrazine hydrate (1.3 mL 1.5 equiv.) is added. This mixture is stirred for 5 h at 70 °C. This reaction mixture is left standing at 0 °C for some time and the precipitate is subsequently filtered off, washed with cold methanol, then washed with ether and dried in vacuum at 45 °C. The structure of the gluconohydrazide was confirmed by ^1^H NMR (Figure S1).

^1^H NMR, DMSO, 500 MHz (δ in ppm): 8.7 (S, 1H), 5.21–5.25 (d, *J* = 5.4 Hz, 1H), 4.45–4.55 (dd, *J* = 5.4 Hz, 1H), 4.30–4.40 (dd, 2H), 4.21 (S, 2H) 3.48–3.91 (m, 1H) 3.99–4.05 (t, 1H).

### 4.3. Preparation of Alkyl Isocyanate from Alkyl Acyl Azide

Palmitoyl chloride (2 g, 1 equiv.) was added to 50 mL of acetone in a double neck RB and sodium azide (0.68 g, 1.45 equiv.), dissolved in 20 mL of water, was added dropwise to the acyl chloride mixture while maintaining a temperature of 10–15 °C. The reaction was continued up to 2 h at room temperature, and an organic layer containing acyl azide was separated. To this azide, benzene was added and refluxed at 80 °C for 1 h. The course of the reaction was followed by infrared analysis. The appearance of the isocyanate peak at 2264 cm^−1^ and the disappearance of the azide peak around 2142 cm^−1^ confirmed the formation of the corresponding isocyanate.

### 4.4. Synthesis of Amphiphilic Glucono Semicarbazide Gelator

**Figure 7:**
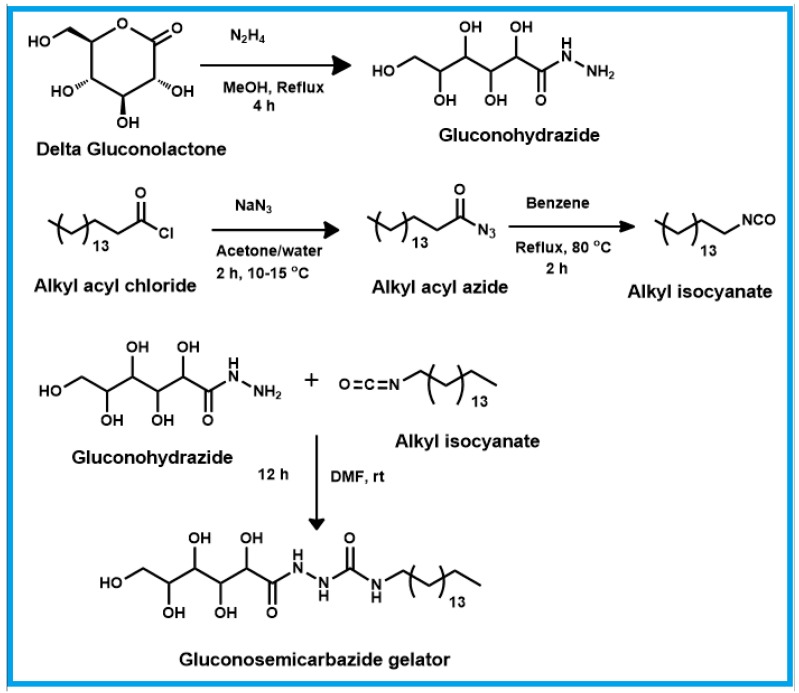
Schematic route to synthesize gluconosemicarbazide gelator.

Alkyl isocyanate (1 g, 1 equiv.) dissolved in 20 mL of anhydrous DMF was added to a double neck RB. Gluconohydrazide (1.4 g, 1.2 equiv.) dissolved in 10 mL of anhydrous DMF was added to the reaction mixture and stirred at 60 °C for 2 h. The reaction was monitored by IR for the disappearance of the isocyanate peak. The reaction mixture was then poured into water and the precipitate was filtered and vacuum-dried at 50 °C overnight. Schematic representation for the synthesis of gluconosemicarbazide gelator is shown in Figure 7. The structure of the compound was confirmed by ^1^H NMR and ^13^C NMR (Figures S2 and S3). 

^1^H NMR, DMSO, 500 MHz (δ in ppm): 9.23 (S, 1H), 7.66 (S, 1H), 5.35–5.39 (d, *J* = 5.3 Hz ,1H), 4.66–4.73 (dd, *J* = 5.3 Hz,1H), 4.44–4.56 (dd, *J* = 5.4 Hz, 2H), 4.24–4.29 (t, 1H), 4.08–4.12 (t, 1H), 3.94–4.03 (m, 1H) 2.92–3.02 (m, 2H), 1.33–1.43 (m, 2H), 1.19–1.32 (m, 24H), 0.82–0.88 (t, 3H). 

^13^C NMR, DMSO, 300 MHz (δ in ppm): 171 (C6), 157 (C7), 72.3 (C2), 71.2 (C5), 71.1 (C4), 70.4 (C3), 63 (C1), 30.8 (C8), 29.2 (C12), 28.6 (C9), 28.55 (C11), 28.2, 25.9 (C10), 21.6 (C13), 13.4 (C14).

### 4.5. Gelation Tests

To confirm, gelation test tube inversion method was employed. A weighed amount of the gelator was added to a mixture of organic solvents (polar aprotic) such as DMSO, NMP, DMF and water in a test tube vial and subjected to heat at an elevated temperature to obtain a homogenous solution. This hot solution, upon cooling, turned to a solid aggregate mass and was found to be stable upon inverting the test tube. Further, the melting temperature of the gels (*T*_gel_) was determined by the dropping ball method, wherein steel balls were placed on the surface of the gels held in a test tube and heated at 1 °C/min using a heating block mantle. The temperature at which the ball reached the bottom of test tube was considered as the gel melting temperature.

In addition, the compound showed gelation when the mixture of organic solvents and water was subjected to sonication at 30 °C for a period of 4–5 min.

### 4.6. Instrumentation

#### 4.6.1. Proton NMR

^1^H NMR spectra was recorded in DMSO-d6 on a BRUKER 500 MHz NMR spectrometer (Bremen, Germany) and tetramethylsilane (TMS) was used as an internal reference. 

#### 4.6.2. FT-IR Spectroscopy

FT-IR measurements on gels prepared from a DMSO/H_2_O mixture and the nature of the gelator-solvent interaction was determined by IR spectroscopy using a Perkin Elmer, spectrum 100 FT-IR spectrometer (PerkinElmer, Waltham, MA, USA). The gel and the solution of the gelator in DMSO/H_2_O solvent were taken on KBr discs and scanned in the 4000–400 cm^−1^ range.

#### 4.6.3. Differential Scanning Calorimetry

The gel melting temperatures were determined by DSC Q 100 series (TA instruments, New Castle, DE, USA) from room temperature to 100 °C at a heating rate of 5 °C/min under a nitrogen atmosphere (flow rate 50 mL/min). Weighed quantity of the gel (12 mg) was taken in a hermitic pan and sealed.

#### 4.6.4. X-ray Diffraction

The xerogels for XRD were obtained by freeze-drying the gels at −100 °C. X-ray diffraction spectra for the xerogels were obtained by using a Siemens/D-5000 X-ray diffractometer (Munich, Germany) using Cu Ka radiation of wavelength 1.54 Å and a continuous scan speed of 0.045/min. Diffraction data were recorded at room temperature, in the range of 2° ≤ 2*θ* ≤ 65°.

#### 4.6.5. Scanning Electron Microscopy (SEM)

SEM images of the xerogels were taken on a Scanning Electron Microscope-Energy Dispersive Spectrometer (SEM-EDS), HITACHI S-3400N (Tokyo, Japan). The accelerating voltage was 15 kV, and the emission was 10 Ma.

#### 4.6.6. Oscillatory Rheological Measurements

Rheological measurements were performed with a strain-controlled rheometer (series Modular Compact Rheometer 102, Anton Paar’s, Graz, Austria-Europe) equipped with a parallel plate. The gap distance was fixed at 1 mm. To determine the storage moduli, *G*′, and loss moduli, *G*″, the measured angular frequency ω, ranged from 0.1 to 100 s^−1^ and the applied shear strain amplitude was 0.5% in the linear viscoelastic regime measured at room temperature (25 °C), at the same time the strain amplitude sweep was performed at a constant frequency (*f*) 1.0 Hz with strain ranging from 0.01 to 100, at 25 °C.

## Supplementary Materials

The following are available online at www.mdpi.com/2310-2861/3/2/12/s1. Figure S1. ^1^H NMR spectrum of gluconohydrazide gelator. Figure S2. ^1^H NMR spectrum of gluconosemicarbazide gelator. Figure S3. ^13^C NMR spectrum of gluconosemicarbazide gelator in DMSO-d_6_. Table S1. Gelation properties of C_16_ gelator [solvent:water = 80:20 (*v*/*v*)].
