# Supplementary material for: Ultrasound- and Temperature-Induced Gelation of Gluconosemicarbazide Gelator in DMSO and Water Mixtures
Source: Gels. 2017 Apr 18;3(2):12. doi: 10.3390/gels3020012 (PMC6318682; doi:10.3390/gels3020012)
Supplement: Supplementary file 1 [file gels-03-00012-s001.pdf]

Ultrasound- and Temperature-Induced Gelation of Gluconosemicarbazide Gelator in DMSO and Water Mixtures

Himabindu M. and Aruna Palanisamy

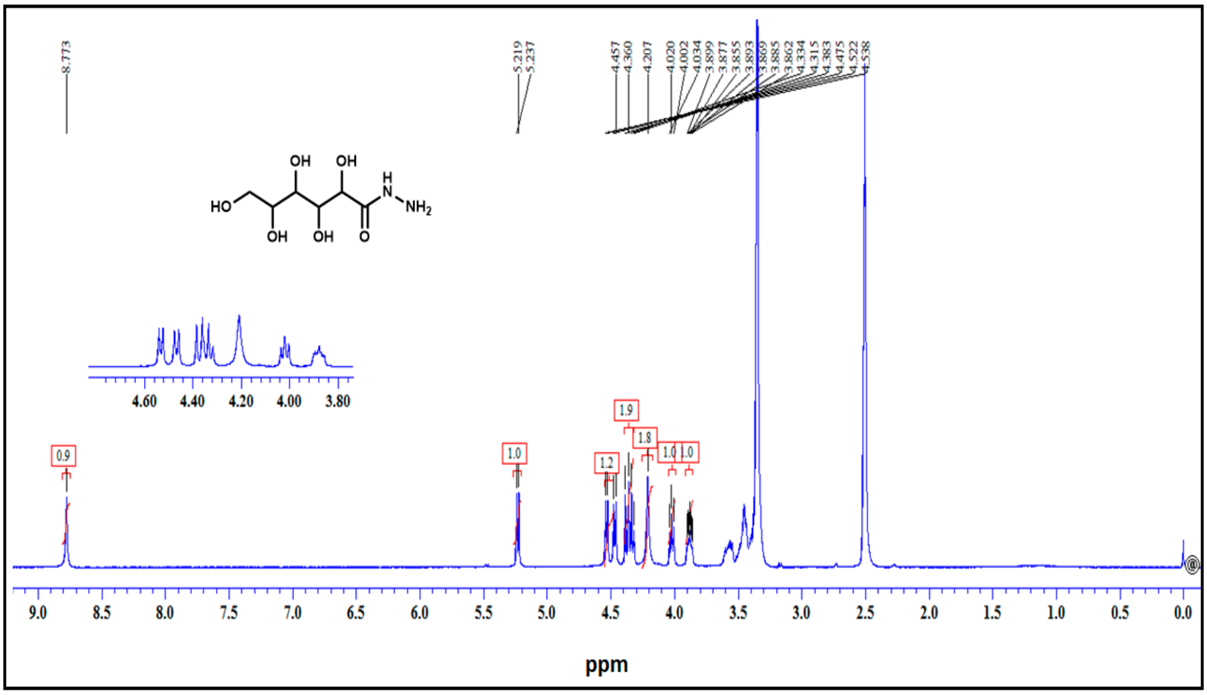

Figure S1. ^{1}H NMR spectrum of gluconohydrazide gelator.

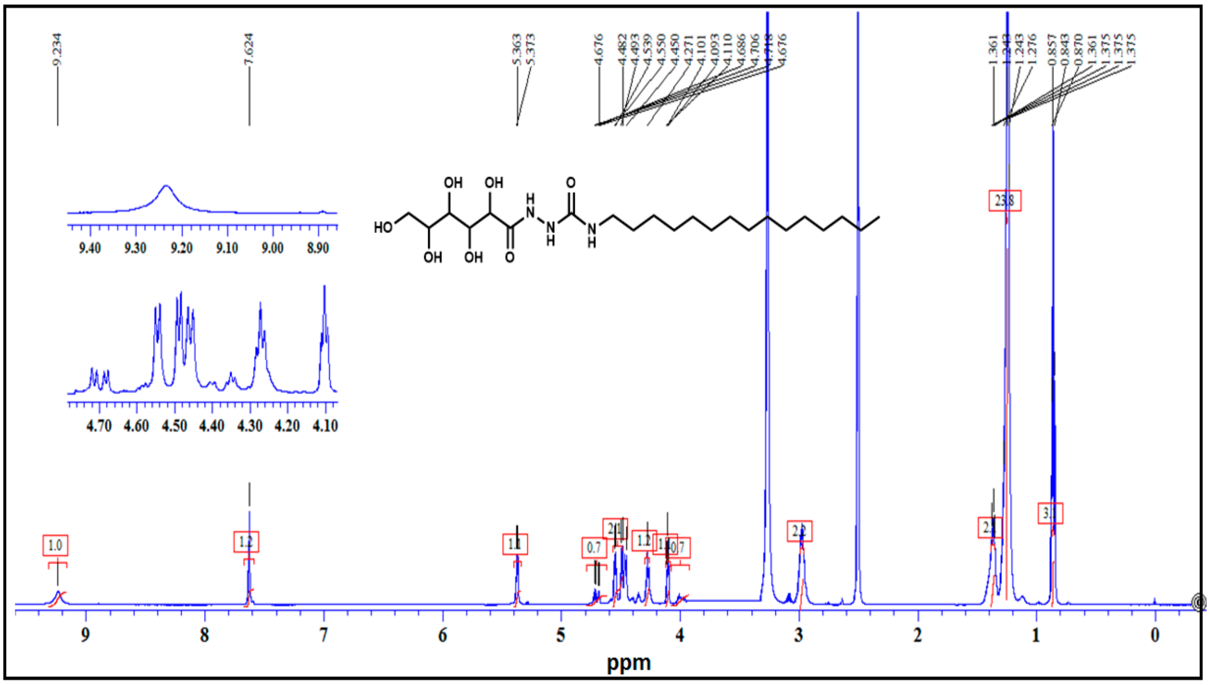

Figure S2. ^{1}H NMR spectrum of gluconosemicarbazide gelator.

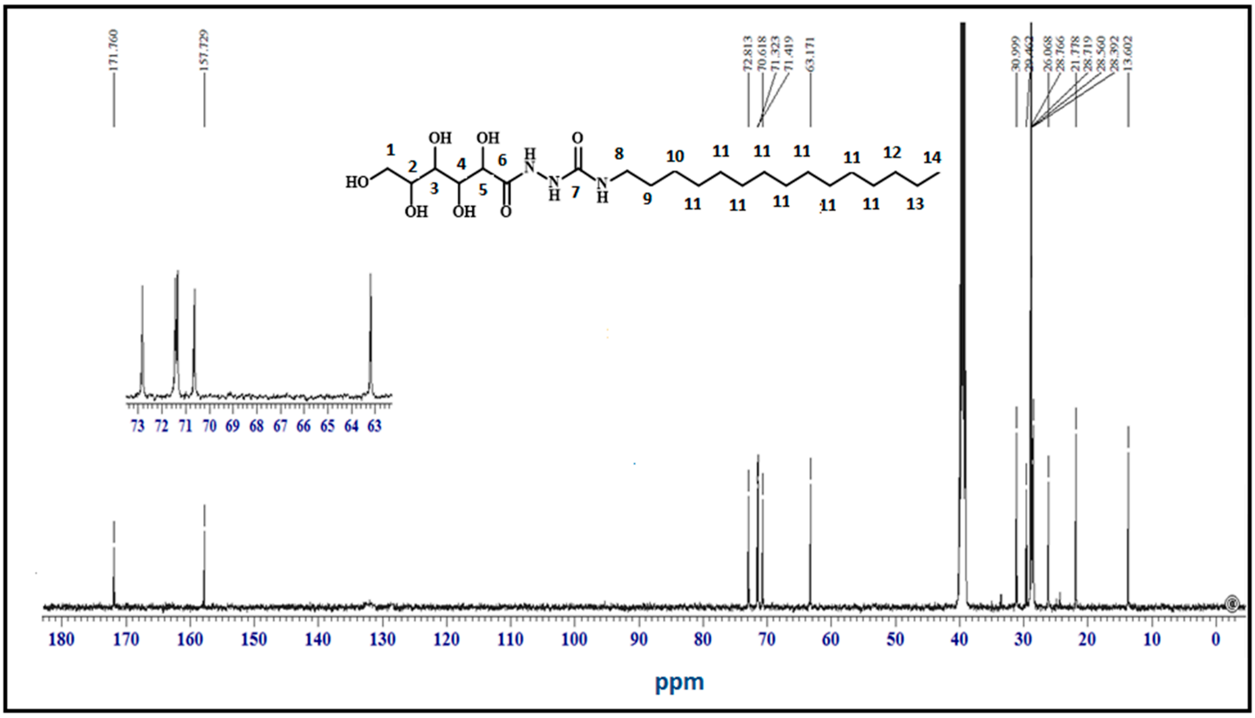

Figure S3. ^{13}C NMR spectrum of gluconosemicarbazide gelator in DMSO-d_6.

Table S1. Gelation properties of C_{16} gelator [solvent:water = 80:20 (*v*/*v*)].

Serial No	Medium	MGC (mg/mL)	Time of Gelation (min)
1	DMSO + H_2O	25	30
2	NMP + H_2O	20	30
3	DMF + H_2O	25	30
4	DMSO	60	360
5	DMAC + H_2O	30	50
6	Buffer PH-5.5 *	30	25
7	Buffer PH-7.4 *	32	120
8	Buffer PH-8.0 *	30	40
9	Buffer PH-12 *	35	30

DMSO, dimethyl sulfoxide; DMF, dimethyl formamide; NMP, *N*-methyl pyrrolidone; DMAc, *N*,*N*-dimethyl acetamide; DMF, *N*,*N*-dimethyl formamide. * Buffer solutions and their composition:

Code	Composition
Buffer PH-5.5	100 mL of 0.1 M Potassium hydrogenphthalate + 73.2 mL of 0.1 M sodium hydroxe
Buffer PH-7.4	100 mL of 0.1 M potassium hydrogenphosphate + 78.2 mL of 0.1 M sodium hydroxide
Buffer PH-8.0	100 mL of 0.1 M potassium hydrogenphosphate + 93.4 mL of 1 M sodium hydroxide
Buffer PH-12	100 mL of 0.05 M disodium hydrogenphosphate + 53.8 mL of 0.1 M of sodium hydroxide
